# The Friendly Societies' Conferences

**Published:** 1914-07-11

**Authors:** 


### NATIONAL INSURANCE ACT DEVELOPMENTS.

### The Friendly Societies' Conferences.

In various parts of the country during Whitsuntide the great Friendly Societies held their annual conferences. To all interested in State Insurance these meetings are of considerable importance, since the views there expressed may be taken as representing the general opinion of Friendly Society members throughout the land; the meetings are attended by delegates from all the branches of the Orders, and the conferences are conducted on democratic lines. Moreover, Friendly Societies have taken such a great part in the administration of the National Insurance Acts, and the experience of these bodies was used to such a large extent as the basis of the whole National Insurance scheme, that the opinions of those intimately associated with the working of the Friendly Societies (especially the large Orders) are worthy of full consideration. It will not, therefore, be out of place if we pay some attention to the proceedings at these conferences which have recently taken place.

### General Opinion on the Acts.

This year's conferences are of more than usual interest, inasmuch as the societies have now had a full year's experience of the administration of benefits, and are in a better position to judge the situation as a whole than they were last year. Broadly speaking, it may be said that since the meetings of a year ago the Friendly Society view of the Acts has undergone little change. Active opposition is, of course, non-existent, the general feeling being that the inevitable must be accepted, and the best must be made of things as they are. The main complaints, as before, are against the complexity of the provisions of the Acts, the multitudinous regulations issued by the Commissioners, and the control and interference instituted by the State. The trouble arises, of course, from the fact that the societies anticipated that they would be able to administer State Insurance in very much the same manner as the previous voluntary insurance, and their hopes in this connection have been entirely falsified. Somewhat bitter complaints were made with regard to the "blow at independence" brought about by the institution of National Insurance, although opinion is universally against the introduction of anything like a voluntary scheme. The Grand Master of the Manchester Unity of Oddfellows, Mr. W. P. Wright, in his presidential address, laid particular stress upon this loss of independence; but it is interesting to note that he attached the principal blame for this state of affairs on the members of Approved Societies themselves. He lamented the fact that, so far as one-half of the insured population is concerned, the section of the Act which provides that the affairs of Approved Societies shall be subject to the absolute control of the members has become a dead letter, and charged the Approved Societies promoted by industrial assurance companies with the denial of the rights and privileges in this direction which the Act was understood to confer. He pointed out, however, that those insured persons who have attached themselves to these societies show not the slightest dissatisfaction at their position, and that, even in those Approved Societies, like the Manchester Unity, where the fullest and most complete rights of self-government have been accorded to members insured for State benefits only, such members show not the slightest desire to avail themselves of these rights. This is certainly a disappointing state of affairs, as it tends to make Approved Societies merely commercial concerns instead of possessing the democratic and thrift-encouraging qualities of the old Friendly Societies.

### The Possibility of Entire State Control.

Another passage of the presidential address of Mr. W. P. Wright is worthy of note. Discussing the Act in general, he said: "I am not ashamed frankly to confess that if I could have had my time over again I would have opposed the State recognition of voluntary organisations, and would have urged that the State, having taken the responsibility of launching a scheme, should have taken upon itself the further responsibility of administering that scheme." The most effective argument that he brings forward in support of this view is the lack of uniformity that at present exists, owing to the fact that the benefits of the Acts are administered by nearly 24,000 different societies in almost as many different ways. It will be remembered that this aspect was emphasised in the Report of the Committee appointed by the Fabian Society, recently reviewed in these columns. The unsatisfactory administration by Insurance Committees was also touched upon and pointed to as a still further hindrance to the attainment of that uniformity which is, for many reasons, so desirable. The arguments thus put forward are certainly sound, but it is doubtful whether the institution of entire State control would be a satisfactory solution of the problem. State control would mean the disappearance of the last fragment of that independence on which the Friendly Societies set such store, and it is really doubtful whether there would be much gain in either efficiency or economy of administration; at any rate present experience of State Departments does not encourage that hope. Uniformity certainly might be secured, but there are other evils than lack of uniformity which might arise under full State control.

With regard to the probability of State administration Mr. W. P. Wright expressed the view that everything was pointing in this direction. He intimated that it was the intention of the Commissioners to set up one or more Approved Societies, under their own management, for deposit contributors and for members of dissolved societies, and pointed out that this is clearly the thin end of the wedge. Whether these prognostications as to the advent of full State control, or even as to the intentions of the Commissioners, are justified remains to be seen, but we hardly think that the State Approved Society is so near as has been suggested.

Centralisation for State Insurance Purposes.

The one great fact emphasised at the conferences of 1913 was that the majority of Friendly Society secretaries were, for many reasons, incompetent efficiently to carry out the requirements of the Acts. This has been increasingly demonstrated in the year that has just gone; and realising that this is a state of affairs that cannot continue, many of the large Orders—notably the National Independent Order of Oddfellows—are taking steps to consolidate their Lodges, in order that the State Insurance benefits may be more effectively administered. Consolidation into sufficiently large groups would enable a whole-time secretary to be appointed and a reasonable salary paid, and the difficulty of requiring more efficient service from already overworked secretaries whose salaries are merely nominal, or who are working in an honorary capacity, would thus be obviated. Alternatively, of course, the centralisation could be carried to its limit, and the whole secretarial work of the Order done at one central office. The administrative machinery of the large Friendly Society Orders would then, so far as State Insurance is concerned, resemble in some respects that of the Approved Societies connected with the large industrial assurance companies. It is doubtful, however, whether such complete centralisation would be so acceptable to the members, and in any case it is possible that the Commissioners would oppose such a step.

The adoption of these schemes of consolidation is indicative of the change of attitude that has taken place in many quarters. At the last conferences the general opinion seemed to be that the Act imposed unnecessarily heavy burdens upon the secretaries, and that the regulations were unduly complicated. This view has somewhat changed, in that it now appears to be recognised that the regulations are necessary if public money is to be scrupulously administered; and although the incompetence of secretaries is still discussed, yet the blame is no longer laid so heavily upon the Insurance Act but upon the existing system itself.

The Effect on Voluntary Insurance.

Turning to the private organisation of the Friendly Societies, the published accounts show that all the more important societies have gained in the number of their members. The introduction to sickness insurance effected by the passing of the National Insurance Acts has brought the advantages of such insurance to the notice of those who would otherwise not have recognised it, and this has resulted in many insured persons voluntarily insuring for benefits in addition to those provided by the State. To this extent, at any rate, the passing of the Acts has been a direct encouragement to thrift, although the Friendly Societies claim that the accession of members has been discounted by disadvantages in other directions. To take one instance, the relief from liability through the operation of Section 72 of the Act of 1911, by virtue of which existing Friendly Society members were to be given the option of reducing their voluntary contributions to the extent of the State contribution, and suffering a corresponding decrease in benefit, has not been anything like so great as was anticipated, the great majority of the members preferring to pay both State and voluntary contributions. The result has been that the double insurance has caused many members to be over-insured, and the rate of claims for sickness benefit has correspondingly increased.

Taking things altogether, however, it may be said that the Friendly Societies have recognised that compulsory National Insurance has come to stay, and are, therefore, directing their attention to securing improvements in administration and in adjusting benefit and contribution arrangements.

